# A Comprehensive Assessment of Vascular and Nonvascular Risk Factors Associated with Migraine

**DOI:** 10.7759/cureus.6189

**Published:** 2019-11-18

**Authors:** Urvish K Patel, Dhaivat Shah, Preeti Malik, Maryam Hussain, Bindi Chauhan, Deepkumar Patel, Shivani Sharma, Nashmia Khan, Kulin Patel, Ashish Kapoor, Tapan Kavi

**Affiliations:** 1 Neurology and Public Health, Icahn School of Medicine at Mount Sinai, New York, USA; 2 Clinical Research, Icahn School of Medicine at Mount Sinai, New York, USA; 3 Pediatrics, The Children's Hospital at Montefiore, Bronx, USA; 4 Public Health, Icahn School of Medicine at Mount Sinai, New York, USA; 5 Public Health, Long Island University, New York, USA; 6 Public Health, New York Medical College, School of Health Science and Practice, Valhalla, USA; 7 Internal Medicine, SUNY Downstate Medical Center, Brooklyn, USA; 8 Internal Medicine, Multicare Tacoma General Hospital, Tacoma, USA; 9 Nursing, Holy Family University, School of Nursing, Philadelphia, USA; 10 Neurology, Bayonne Medical Center‑ Carepoint Health & Jersey City Medical Center‑ RWJ Barnabas Health, Jersey City, USA; 11 Neurology, Cooper Neurological Institute, Cooper University Hospital, Camden, USA

**Keywords:** migraine, risk factors, vascular disease, stroke, hypertension, nationwide inpatient sample, obesity, vitamin d deficiency, substance abuse, tobacco

## Abstract

Introduction: Migraine is a chronic disabling neurological disease, with an estimated expense of $15-20 million/year. Several studies with a small number of patients have studied risk factors for migraine such as cardiovascular disorders, stroke, smoking, demographic, and genetic factors but this is the first comprehensive study for evaluation of vascular and nonvascular risk factors. It is important to evaluate all the risk factors that help to prevent the healthcare burden related to migraine.

Methodology: We performed a retrospective cross-sectional analysis of the Nationwide Inpatient Sample (NIS) (years 2013-2014) in adult (>18-years old) hospitalizations in the United States. Migraine patients were identified using ICD-9-CM code to determine the demographic characteristics, vascular, and nonvascular risk factors. Univariate analysis was performed using the chi-square test and a multivariate survey logistic regression analysis was performed to identify the prevalence of the risk factors and evaluate the odds of prevalence of risk factors amongst migraine patients compared to nonmigraine patients, respectively.

Results: On weighted analysis, after removing missing data of age, gender and race, from years 2013 to 2014, of the total 983,065 (1.74%) migraine patients were identified. We found that younger (median age 48-years vs. 60-years), female (82.1% vs. 58.5%; p<0.0001), white population (76.8% vs. 70.5%; p<0.0001), and privately insured (41.1% vs. 27.4%; p<0.0001) patients were more likely to have migraine than others. Cerebral atherosclerosis, diabetes mellitus, ischemic heart disease, atrial fibrillation, and alcohol abuse were not significantly associated with migraine. Migraineurs had higher odds of having hypertension [odds ratio (OR): 1.44; 95% confidence interval (CI): 1.43-1.46; 44.49% vs. 52.84%], recent transient ischemic attack (TIA) (OR: 3.13; 95%CI: 3.02-3.25; 1.74% vs. 0.67%), ischemic stroke (OR: 1.40; 95%CI: 1.35-1.45; 2.06% vs. 1.97%), hemorrhagic stroke (OR: 1.11; 95%CI: 1.04-1.19; 0.49% vs. 0.46%), obesity (OR: 1.46; 95%CI: 1.44-1.48; 19.20% vs. 13.56%), hypercholesterolemia (OR: 1.33; 95%CI: 1.30-1.36; 5.75% vs. 5.54%), substance abuse (OR: 1.51; 95%CI: 1.48-1.54; 7.88% vs. 4.88%), past or current consumption of tobacco (OR: 1.40; 95%CI: 1.38-1.41; 31.02% vs. 27.39%), AIDS (OR: 1.13; 95%CI: 1.04-1.24; 0.33% vs. 0.41%), hypocalcemia (OR: 1.09; 95%CI: 1.03-1.14; 0.77% vs. 0.89%), and vitamin D deficiency (OR: 1.93; 95%CI: 1.88-1.99; 2.47% vs. 1.37%) than patients without migraine. Female patients were at a higher risk of migraine (OR: 3.02; 95%CI: 2.98-3.05) than male.

Conclusion: In this study, we have identified significant risk factors for migraine hospitalizations. Early identification of these risk factors may improve the risk stratification in migraine patients.

## Introduction

Migraine is a primary headache disorder that impacts about one-fifth of the general population of the United States, and females are three to four times more frequently affected than males [1-3]. Estimates of the incidence of chronic migraine worldwide vary from 0% to 5.1%, with most general population surveys reporting a prevalence of 1.4%-2.2% [4]. Migraine impacts 12% of adults in the United States, making this disease a predominant concern for many patients and their physicians [5]. Thus, the disability caused by diseases such as migraine has become a major cause of the global burden of public health and socioeconomic expenses.

Migraine often affects people during their most productive years of life, exerts substantive individual and societal costs, causing a considerable degree of disability and is associated with several risk factors and comorbidities [6]. The largest incidence is among females between the ages of 18 and 49 [4]. Treatment includes avoidance of migraine triggers and risk factor modification, as well as drug and nondrug-based treatments to prevent and abort migraine attacks. The recognition and management of disorders and risk factors that are comorbid with chronic migraine optimizes patient outcomes [7].

It is important to identify the full spectrum of risk factors to help prevent migraine hospitalizations. Several studies have looked at risk factors such as cardiovascular disorders, stroke, fibromyalgia, smoking, vitamin D deficiency, demographic and genetic factors [7-11], but a comprehensive study like this has not been done before. Due to the high prevalence of migraine, any association between migraine and vascular and nonvascular disease could have a substantial effect on public health.

## Materials and methods

Data were obtained from the Agency for Healthcare Research and Quality's Healthcare Cost and Utilization Project (HCUP) Nationwide Inpatient Sample (NIS) between January 2013 and December 2014. The NIS is the largest publicly available all-payer inpatient care database in the United States and contains discharge-level data provided by states that participate in the HCUP (including a total of 46 in 2011). This administrative dataset contains data on approximately eight million hospitalizations in 1,000 hospitals that were chosen to approximate a 20% stratified sample of all US community hospitals, representing more than 95% of the national population. Discharge weights are provided for each patient discharge record, which allows extrapolation to obtain national estimates. Each hospitalization is treated as an individual entry in the database and is coded with one principal diagnosis, up to 24 secondary diagnoses, and 15 procedural diagnoses associated with that stay. Detailed information on NIS is available at http://www.hcup-us.ahrq.gov/db/nation/nis/nisdde.jsp. The NIS is a de-identified database, so informed consent or Institutional Review Board (IRB) approval was not needed for the study. The HCUP Data Use Agreement (HCUP-348L73IZS) for the data utilized in this study was obtained.

Study population

We used the ninth revision of the International Classification of Diseases, clinical modification code (ICD-9-CM) to identify adult patients admitted to hospital with a primary or secondary diagnosis of migraine (ICD-9-CM code: 346) from all adult hospitalizations of the year 2013-2014. Similarly, patients with concurrent vascular and nonvascular risk factors were identified as secondary diagnosis using ICD-9-CM code. Age <18 years and admissions with missing data for age, gender, and race were excluded. The sample size was based on the available data. Data from NIS have been previously used to identify and analyze the trend, outcomes, healthcare cost, and disparity of care [7-8].

Patient and hospital characteristics

Patient characteristics of interest were age, gender, race, insurance status, and concomitant diagnoses as defined above. The race was defined by White (referent), African American, Hispanic, Asian or Pacific Islander, and Native American. Insurance status was defined by Medicare (referent), Medicaid, Private Insurance, and Other/Self-pay/No charge. We defined the severity of comorbid conditions using Deyo's modification of the Charlson comorbidity index (Table 1).

**Table 1:** Deyo’s modification of Charlson’s comorbidity index.

Condition	ICD-9-CM codes	Charlson score
Myocardial infarction	410-410.9	1
Congestive heart failure	428-428.9	1
Peripheral vascular disease	433.9, 441-441.9, 785.4, V43.4	1
Cerebrovascular disease	430-438	1
Dementia	290-290.9	1
Chronic pulmonary disease	490-496, 500-505, 506.4	1
Rheumatologic disease	710.0, 710.1, 710.4, 714.0-714.2, 714.81, 725	1
Peptic ulcer disease	531-534.9	1
Mild liver disease	571.2, 571.5, 571.6, 571.4-571.49	1
Diabetes	250-250.3, 250.7	1
Diabetes with chronic complications	250.4-250.6	2
Hemiplegia or paraplegia	344.1, 342-342.9	2
Renal disease	582-582.9, 583-583.7, 585, 586, 588-588.9	2
Any malignancy including leukemia and lymphoma	140-172.9, 174-195.8, 200-208.9	2
Moderate or severe liver disease	572.2-572.8	3
Metastatic solid tumor	196-199.1	6
AIDS	042-044.9	6

Outcomes

The primary outcome of interest was to assess the vascular and nonvascular risk factors associated with migraine amongst patients admitted between January 2013 and December 2014 US hospitalizations.

Statistical analysis

All statistical analyses were performed using the weighted survey methods in SAS (version 9.4). Weighted values of patient-level observations were generated to produce a nationally representative estimate of the entire US population of hospitalized patients. A p-value of <0.05 was considered significant. Univariate analysis of differences between categorical variables was tested using the chi-square test and analysis of differences between the continuous variable (age) was tested using paired Student's t-test. Mixed-effects survey logistic regression models with weighted analysis were used for categorical dependent variables in order to estimate odds ratio (OR) and 95% confidence interval (CI) for prediction of vascular and nonvascular risk factors associated with migraine in the year 2013-2014. We included demographics (age, gender, race), patient-level hospitalization variables (admission day, primary payer, admission type, median household income category), hospital-level variables (hospital region, teaching versus nonteaching hospital, hospital bed size), and Charlson’s comorbidity index (CCI).

For each model, c- index (a measure of goodness of fit for binary outcomes in a logistic regression model) was calculated. All statistical tests used were two-sided, and p<0.05 was deemed statistically significant. No statistical power calculation was conducted prior to the study.

## Results

Disease hospitalizations

From the year 2013 to 2014, after excluding patients with aged <18 years and admissions with missing data for age, gender, and race, we found a total of 56,499,788 nationwide inpatient adult hospitalizations. Out of them, a total of 983,065 (1.74%) patients had migraine.

Demographics, patient and hospital characteristics, and comorbidities

Among total hospitalized adult patients, higher prevalence of migraine was observed in patients of younger age group (median age: 48-years vs. 60-years; p<0.0001), females (82.11% vs. 58.52%; p<0.0001), White population (76.84% vs. 70.51%; p<0.0001), and patients with private insurance (41.13% vs. 27.44%; p<0.0001) compared to others (Table 2).

**Table 2:** Characteristics of migraine patients in nationwide inpatient adult hospitalizations. The percentages here are column % that indicates direct comparison of characteristics between migraineurs and non-migraineurs amongst nationwide inpatient hospitalizations. *Bedsize of hospital indicates the number of hospital beds which varies depending on hospital location (Rural/ Urban), teaching status (Teaching/Nonteaching) and Region (Northeast/Midwest/Southern/Western).

	Migraine	No migraine	Total	p-value
Inpatient adult hospitalization weighted (%)	983065 (1.74%)	55516723 (98.26%)	56499788 (100%)	<0.0001
Demographics of patients				
Age median (SD) (years)	48 (16)	60 (21)		
Gender (%)				<0.0001
Male	17.89%	41.48%	41.07%	
Female	82.11%	58.52%	58.93%	
Race (%)				<0.0001
White	76.84%	70.51%	70.62%	
African American	12.76%	15.25%	15.21%	
Hispanic	8.61%	11.00%	10.96%	
Asian or Pacific Islander	1.22%	2.62%	2.60%	
Native American	0.57%	0.61%	0.61%	
Characteristics of patients				
Median household income category for patient's zip code (%)				<0.0001
0-25th percentile	26.79%	30.21%	30.15%	
26-50th percentile	26.83%	26.75%	26.75%	
51-75th percentile	24.47%	23.19%	23.21%	
76-100th percentile	21.91%	19.85%	19.88%	
Primary payer (%)				<0.0001
Medicare	30.18%	47.03%	46.73%	
Medicaid	0.33%	16.86%	16.90%	
Private insurance	41.13%	27.44%	27.68%	
Other/self-pay/no charge	9.53%	8.67%	8.69%	
Admission type (%)				0.0445
Nonelective	75.38%	75.47%	75.47%	
Elective	24.62%	24.53%	24.53%	
Admission day (%)				<0.0001
Weekday	80.98%	79.69%	79.71%	
Weekend	19.02%	20.31%	20.29%	
Characteristics of hospitals				
Bedsize of hospital (%) *				<0.0001
Small	15.45%	16.23%	16.22%	
Medium	27.54%	28.06%	28.05%	
Large	57.01%	55.71%	55.73%	
Hospital location and teaching status (%)				<0.0001
Rural	7.80%	10.29%	10.25%	
Urban non-teaching	31.28%	33.33%	33.29%	
Urban Teaching	60.92%	56.39%	56.47%	
Hospital region (%)				<0.0001
Northeast	19.46%	20.11%	20.10%	
Midwest	21.79%	20.47%	20.49%	
South	39.68%	40.14%	40.13%	
West	19.07%	19.27%	19.27%	
Deyo's Charlson comorbidity index (CCI)				<0.0001
0	47.53%	41.87%	41.96%	
1	27.92%	21.37%	21.48%	
2	12.21%	13.48%	13.46%	
3	5.63%	8.42%	8.37%	
4	2.70%	5.77%	5.72%	
5	4.00%	9.09%	9.00%	

The risk factors associated with migraine

Table 3 has mentioned the prevalence of vascular and nonvascular risk factors associated with migraine amongst total adult hospitalizations. There was a higher prevalence of transient ischemic attack (1.74% vs. 0.67%; p<0.0001), acute ischemic stroke (2.06% vs. 1.97%; p<0.0001), hemorrhagic stroke (0.49% vs. 0.46%; p<0.0001), hypercholesterolemia (5.75% vs. 5.54%; p<0.0001), obesity (19.20% vs. 13.56%; p<0.0001), substance abuse (7.88% vs. 4.88; p<0.0001), current or past smoking history (31.02% vs. 27.39%; p<0.0001), and vitamin D deficiency (2.47% vs. 1.37%; p<0.0001) in migraineurs compared to nonmigraineurs. 

**Table 3:** Univariate analysis of vascular and nonvascular risk factors associated with migraine. The percentages here are column % that indicates a direct comparison of risk factors between migraineurs and nonmigraineurs amongst nationwide inpatient hospitalizations.

	Migraine	No migraine	Total	p-value
Inpatient adult hospitalization weighted (%)	983065 (1.74%)	55516723 (98.26%)	56499788 (100)	<0.0001
Vascular risk factors				
Diabetes	17.27%	25.57%	25.42%	<0.0001
Hypertension	44.49%	52.84%	52.69%	<0.0001
Transient ischemic attack	1.74%	0.67%	0.69%	<0.0001
Acute ischemic stroke	2.06%	1.97%	1.97%	<0.0001
Hemorrhagic stroke	0.49%	0.46%	0.46%	<0.0001
Obesity	19.20%	13.56%	13.66%	<0.0001
Hypercholesterolemia	5.75%	5.54%	5.54%	<0.0001
Cerebral atherosclerosis	0.21%	0.41%	0.41%	<0.0001
Ischemic heart diseases	10.94%	21.69%	21.50%	<0.0001
Atrial fibrillation	4.81%	13.02%	12.88%	<0.0001
Nonvascular risk factors				
Substance abuse	7.88%	4.88%	4.93%	<0.0001
Alcohol abuse	4.12%	6.01%	5.98%	<0.0001
Current or past smoker	31.02%	27.39%	27.45%	<0.0001
AIDS	0.33%	0.41%	0.40%	<0.0001
Renal failure	9.69%	21.00%	20.80%	<0.0001
Hypocalcemia	0.77%	0.89%	0.88%	<0.0001
Vitamin D deficiency	2.47%	1.37%	1.39%	<0.0001

Regression model derivation

We performed the multivariable survey logistic regression model to predict the vascular and nonvascular risk factors associated with migraine after adjusting for basic demographic characteristics with patient and hospital-level variables, and CCI (Table 3). In this multivariate regression analysis, migraineurs had higher odds of having hypertension [adjusted odds ratio (OR): 1.44; 95% CI: 1.43-1.46; p<0.0001], transient ischemic attack [adjusted OR: 3.13; 95%CI: 3.02-3.25; p<0.0001], acute ischemic stroke [adjusted OR: 1.40; 95%CI: 1.35-1.45; p<0.0001], hemorrhagic stroke [adjusted OR: 1.14; 95%CI: 1.04-1.19; p=0.0017], obesity [adjusted OR: 1.46; 95%CI: 1.44-1.48; p<0.0001], hypercholesterolemia [adjusted OR: 1.33; 95%CI: 1.30-1.36; p<0.0001], substance abuse [adjusted OR: 1.51; 95%CI: 1.48-1.54; p<0.0001], current or past smoking history [adjusted OR: 1.40; 95%CI: 1.38-1.41; p<0.0001], AIDS [adjusted OR: 1.13; 95%CI: 1.04-1.24; p=0.0057], hypocalcemia [adjusted OR: 1.09; 95%CI: 1.03-1.14; p=0.0025], and vitamin D deficiency [adjusted OR: 1.93; 95%CI: 1.88-1.99; p<0.0001] compared to nonmigraineurs. Similarly, females had higher odds of having migraine compared to males (adjusted OR: 3.02; 95%CI: 2.98-3.05; p<0.0001) (Table 4).

**Table 4:** Multivariate logistic regression analysis to predict vascular and nonvascular risk factors associated with migraine. UL: upper limit; LL: lower limit The model is adjusted for basic demographic with patient-level variables, comorbidities, CCI, concurrent conditions, and hospital-level variables such as hospital region, teaching status, and bed size.

	Odds ratio	Confidence interval		p-value
		LL	UL	
Age (every 10 years)	0.98	0.98	0.98	<0.0001
Gender				
Male	Reference			
Female	3.02	2.98	3.05	<0.0001
Race				
White	Reference			
African American	0.63	0.62	0.64	<0.0001
Hispanic	0.59	0.58	0.60	<0.0001
Asian or Pacific Islander	0.34	0.33	0.36	<0.0001
Native American	0.79	0.74	0.84	<0.0001
Median household income category for patient's zip code				
0-25th percentile	Reference			
26-50th percentile	1.08	1.07	1.10	<0.0001
51-75th percentile	1.10	1.09	1.12	<0.0001
76-100th percentile	1.17	1.15	1.18	<0.0001
Primary payer				
Medicare	Reference			
Medicaid	0.91	0.89	0.92	<0.0001
Private insurance	1.31	1.29	1.33	<0.0001
Other/self-pay/no charge	1.10	1.08	1.12	<0.0001
Admission type				
Nonelective	Reference			
Elective	0.72	0.72	0.73	<0.0001
Admission day				
Weekday	Reference			
Weekend	0.91	0.90	0.93	<0.0001
Bedsize of hospital				
Small	Reference			
Medium	1.02	1.00	1.03	0.0211
Large	1.08	1.07	1.10	<0.0001
Hospital location & teaching status				
Rural	Reference			
Urban nonteaching	1.22	1.19	1.24	<0.0001
Urban teaching	1.43	1.40	1.45	<0.0001
Hospital region				
Northeast	Reference			
Midwest	1.05	1.04	1.07	<0.0001
South	1.03	1.02	1.05	<0.0001
West	1.04	1.03	1.06	<0.0001
Vascular risk factors and comorbidities				
Diabetes	0.80	0.79	0.81	<0.0001
Hypertension	1.44	1.43	1.46	<0.0001
Transient ischemic attack	3.13	3.02	3.25	<0.0001
Acute ischemic stroke	1.40	1.35	1.45	<0.0001
Hemorrhagic stroke	1.14	1.04	1.19	0.0017
Obesity	1.46	1.44	1.48	<0.0001
Hypercholesterolemia	1.33	1.30	1.36	<0.0001
Cerebral atherosclerosis	0.75	0.68	0.83	<0.0001
Ischemic heart diseases	0.75	0.74	0.76	<0.0001
Atrial fibrillation	0.57	0.56	0.58	<0.0001
Nonvascular risk factors and comorbidities				
Substance abuse	1.51	1.48	1.54	<0.0001
Alcohol abuse	0.68	0.66	0.69	<0.0001
Current or past smoker	1.40	1.38	1.41	<0.0001
AIDS	1.13	1.04	1.24	0.0057
Renal failure	0.64	0.63	0.65	<0.0001
Hypocalcemia	1.09	1.03	1.14	0.0025
Vitamin D deficiency	1.93	1.88	1.99	<0.0001
Deyo's Charlson comorbidity index (CCI)	0.99	0.99	1.00	0.0003
c-index	0.729			

Accuracy of the model

Adjusted model to predict the vascular and nonvascular risk factors associated with migraine had the c- statistic of 0.729, which is >0.7 indicating a very good model. 

## Discussion

Migraine is a highly burdensome disease and has great deleterious effect on patient's life, their families, and society. Migraine is the leading cause of outpatient and ED visits and is an important public health problem [3]. An analysis of the 2016 Global burden of disease (GBD data estimated that migraine caused 45.1 million years lived with disability (YLDs) in that year [12]). Our study found that 983,065 (1.74%) patients had a migraine, out of total 56,499,788 nationwide inpatient adult hospitalizations from the year 2013 to 2014.

A 2015 review study by Burch et al. [3] used the most recent government statistics in the United States from the National Health Interview Survey (NHIS), the National Ambulatory Medical Care Survey (NAMCS), the National Hospital Ambulatory Medical Care Survey (NHAMCS), and the Defense Medical Surveillance System (DMSS) and reported the highest prevalence of migraine which remained stable over the past eight years, despite different methods and sample size in the above studies. Migraine is affecting approximately 1 out of every 7 Americans annually (14.9% on average). This prevalence is close to the 11.7% migraine prevalence observed in the American Migraine Prevalence and Prevention (AMPP) study [5].

We also found higher prevalence of migraine in females (82.11% vs. 58.52%; p<0.0001) and White population (76.84% vs. 70.51%; p<0.0001). In support of our findings, four large US population-based migraine studies including AMS I, AMS II, AMPP, and CaMEO study have also reported a higher prevalence of migraine in females and whites [5, 10, 13-14].

This study found various vascular and nonvascular risk factors associated with migraine. In our study results, migraineurs had 1.46 times higher odds of obesity compared to nonmigraineurs (p<0.0001). A meta-analysis study by Ornello et al. also reported that obese subjects had an increased risk of having chronic migraine [pooled adjusted effect estimate (PAEE): 1.75; p<0.001] [15]. Similar results are reported by Fava et al. and explained that insulin resistance associated with obesity might increase the risk of chronic migraine [16]. However, the mechanism linking obesity with increased migraine attacks is not clearly understood.

We also found that migraineurs have higher odds of having hypertension (OR: 1.44; p<0.0001). The Gardener et al. study also reported that hypertension is associated with migraine (OR: 1.76, p<0.0001) [17]. Common environmental and lifestyle factors that may be associated with both migraine and hypertension include diet (salty foods), physical inactivity, chronic stress; both are linked to the metabolic syndrome [18]. Stress-related occupation has been linked to migraine, high blood pressure, and more recently ischemic stroke [19].

Important finding of our study is that migraineurs had a higher risk of cerebrovascular disease (CVD) including transient ischemic attack (OR: 3.13; p<0.0001), acute ischemic stroke (OR: 1.40; p<0.0001), and hemorrhagic stroke (OR: 1.14; p=0.0017). Our study results were supported by a 2017 meta-analysis of 11 prospective studies and 2,221,888 patients; they found a relative risk (RR) of 1.64 for ischemic stroke and RR of 1.15 for hemorrhagic stroke among migraineurs compared to nonmigraineurs [20]. The pathogenic mechanisms explaining the association between migraine and stroke are not clearly understood, but a few hypotheses exist. Some suggested hypothetical theories to explain the migraine stroke relation are cortical spreading depression [21], vasoconstriction, endovascular dysfunction [22], increased prevalence of vascular risk factors, shared genetic defects [23], neurogenic inflammation [24], and hypercoagulability [22, 25].

We found that migraineurs are at 1.40 times higher risk of smoking (current/past). Some studies have indicated the positive correlation between cigarette smoking and migraine [9]. Smoking among migraineurs may be related to the development of cranial autonomic symptoms, but clear evidence showing tobacco use increasing the migraine attack is lacking [11].

We found, another major risk associated with a migraine that is, hypocalcemia (OR: 1.09; p=0.00250), and vitamin D deficiency (OR: 1.93; p<0.0001). A study by Patel et al. similarly reported that the odds of having migraine attacks were higher among vitamin D deficiency (OR: 1.97%; p<0.0001) and hypocalcemia (OR: 1.11; p=0.0061) patients [8]. The possible explanation of this association between migraine and Vitamin D deficiency and hypocalcemia can be neurogenic inflammation involved with the migraine cascade of events [26, 27] and the role of vitamin D in increasing the inflammation and the observed phenomenon of neuronal hyperexcitability in hypocalcemia leading to increased severity of the migraine attacks [28].

Strength and limitations

This is the largest population-based study which comprehensively evaluated the vascular and nonvascular risk factors associated with migraine. Despite being a large study with good statistical power, this study has limitations. Data from clinical registries or administrative databases are obtained retrospectively by chart abstractions based on the discharge diagnosis codes, billing codes, etc., and hence susceptible to coding errors. We evaluated the risk factors in migraine patients who were admitted primarily due to migraine-related hospitalization or secondary to other conditions, so these risk factors may or may not be responsible for increasing severity of migraine or status migrainosus.

## Conclusions

In this study, we identified risk factors for migraine hospitalizations. Identification and management of these risk factors are very important to prevent migraine hospitalizations and to reduce the enormous healthcare burden resulting from it.
